# Centromere Clustering and Spindle Organization in *Dictyostelium* Amoebae

**DOI:** 10.3390/cells15161449

**Published:** 2026-08-12

**Authors:** Ralph Gräf, Marianne Grafe, Irene Meyer

**Affiliations:** Department of Cell Biology, University of Potsdam, Karl-Liebknecht-Str. 24-25, 14476 Potsdam-Golm, Germany; mgrafe@uni-potsdam.de (M.G.); irene.meyer@uni-potsdam.de (I.M.)

**Keywords:** centromere, kinetochore, mitosis, centrosome, *Dictyostelium*

## Abstract

The phenomenon of centromere clustering and its impact are still largely unclear in the eukaryotic supergroup of *Amoebozoa*. In this review, we highlight the molecular basis for centromere clustering, its relationship to the centrosome, and its role in mitotic spindle organization in *Dictyostelium* amoebae. Here, the centrosome is anchored to the cytosolic side of the nucleus during interphase and is connected via the nuclear envelope to the nuclear lamina and a cluster of the centromeres of all six chromosomes. Upon transition from the G2 phase to mitosis, the centrosome penetrates a fenestra in the persisting nuclear envelope and duplicates. During this process, microtubule connections are established between the mitotic centrosomes and the kinetochores. We review all known molecular players in this process and pose a hypothesis on how the centromere protein Cenp68, which is related to monopolin from fission yeast, could facilitate and accelerate the process of mitotic spindle formation.

## 1. Introduction

Centromeres are a defined region of the chromatin, at which the two sister chromatids that emerge during S-phase of the cell cycle remain connected until anaphase of mitosis. They also define the sites where the kinetochores assemble during mitosis to allow spindle assembly, checkpoint function, and establishment of stable connections between mitotic spindle microtubules and sister chromatids. The centromeric chromatin is in a heterochromatin state and is characterized by specific repetitive DNA elements, often including retrotransposable elements [1]. Centromeric DNA sequences favor the inclusion of specialized histones, especially the ubiquitous CENP-A (a centromere-specific histone 3) or, in animals, also CENP-T/W/S/X, which form a complex replacing the canonical core histones at centromeres. At the G2/M transition, upon activation of cyclin-dependent kinase 1 (CDK1), kinetochore proteins assemble at the centromeric chromatin [2].

In many cases, centromeres of individual chromosomes are clustered in close proximity also in interphase. Centromere clustering has been observed in numerous organisms of the major eukaryotic groups, including yeasts, plants, insects, and mammals [3]. When centromere clustering is accompanied by a close grouping of telomeres at the opposite hemisphere of the nucleus, this is also referred to as a Rabl configuration [3]. In fungi, centromere clusters are often located close to the nuclear envelope and coupled to the centrosome/spindle pole body (SPB) via the LINC complex (linker of the nucleus and the cytoskeleton) [4,5]. Centromere clustering appears to facilitate spindle formation, since it brings the centromeres, i.e., the assembly platforms for kinetochores, in close vicinity to the spindle pole bodies already prior to mitotic onset [6].

**Figure 1:**
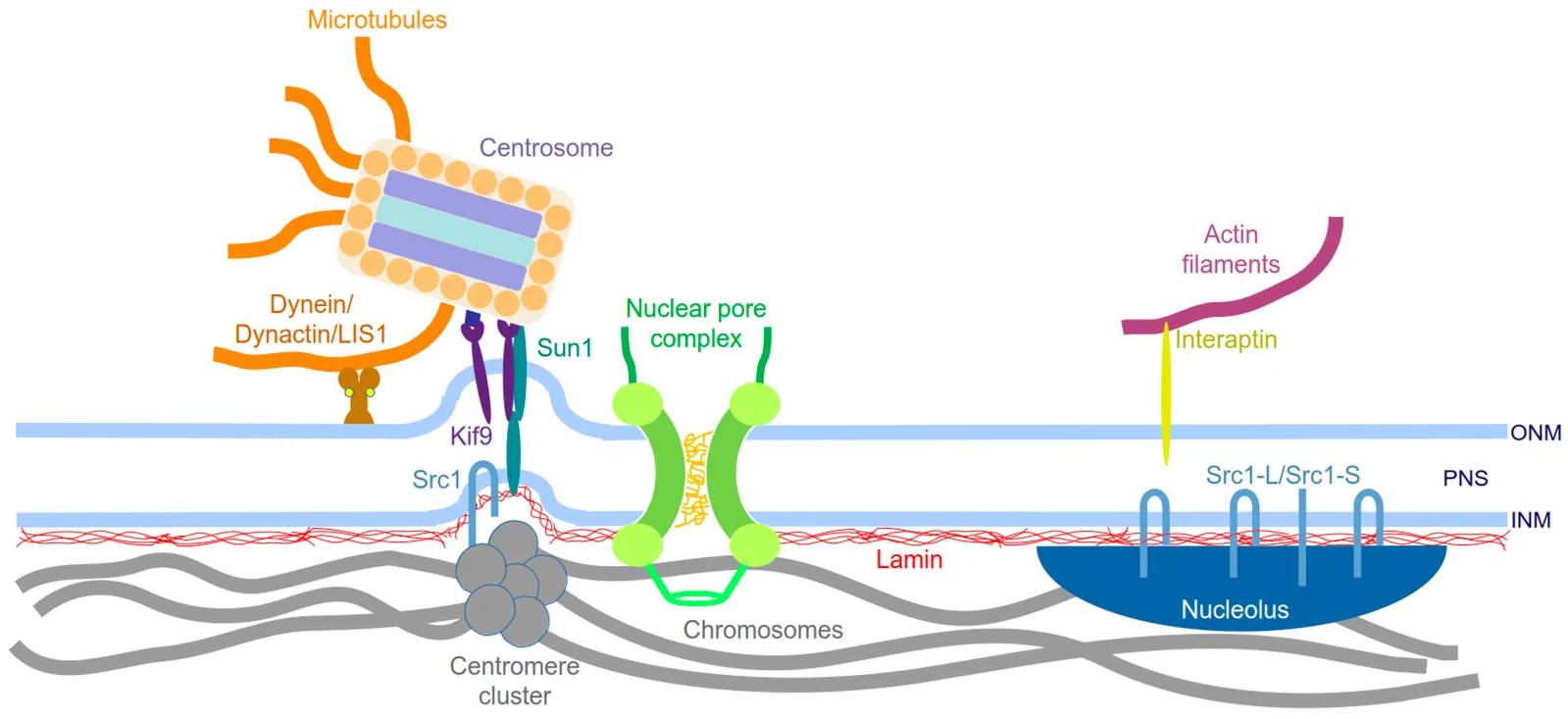
Structural organization of the nuclear envelope of *Dictyostelium* amoebae. Proteins discussed in the text are shown at their known localizations. Abbreviations: ONM, outer nuclear membrane; INM, inner nuclear membrane; PNS, perinuclear space. Modified from ref. [10].

Within the eukaryotic clade of *Amoebozoa*, *Dictyostelium discoideum* is the best-studied model organism. This normally unicellular organism lies on the borderline to multicellularity, since the cells that normally feed on microorganisms emit a cAMP signal when food is scarce; this signal is detected by neighboring cells and triggers chemotaxis. This results in the formation of multicellular clusters, from which stalk cells and spore cells then differentiate to form a fruiting body. The spores survive adverse environmental conditions; once these conditions have passed, amoebae hatch from the spores again. This is why, for decades, *Dictyostelium* amoebae have been one of the favorite models in developmental biology and in studies of signaling and cytoskeletal processes during chemotaxis and cell migration. Their similarity to migratory animal cells also makes them an attractive model for various human diseases [7]. Besides yeasts, *Dictyostelium* amoebae are also one of the best-studied models for an acentriolar centrosome. In most organisms, except higher plants, centrosomes serve as main microtubule organizing centers and as mitotic spindle poles. In *Dictyostelium* amoebae, the centrosome is located on the cytosolic side of the nucleus during interphase and is connected across the nuclear envelope to the nuclear lamina and centromeres, which are gathered in a collective cluster via a Sun1-containing connecting structure [8,9]. Centromeres and centrosomes are therefore located in close proximity also during interphase, separated only by the nuclear envelope (Figure 1).

During mitosis, there is no nuclear envelope breakdown; however, the lamin NE81 disassembles to allow nuclear envelope constrictions during karyokinesis [11]. Permeabilization of the nuclear envelope for access to spindle assembly factors is achieved via partial disassembly of nuclear pore complexes [12,13]. During the transition from the G2 phase to mitosis, the centrosome penetrates a window in the nuclear envelope and duplicates, while microtubule connections are established between the mitotic centrosomes and the kinetochores. The topological relationships between the centrosome and centromeres and the course of mitosis suggest that the purpose of centromere clustering is to ensure that the centromeres, at which the kinetochores assemble during the G2/M transition, are already located in close proximity to the mitotic centrosomes before they duplicate and organize spindle microtubules. Therefore, there is no obvious need for a spindle formation mechanism such as in mammalian cells, where Ran-dependent spindle formation starts from the chromatin and augmentation of spindle microtubules via augmin supports the search-and-capture mechanism of spindle microtubules emanating from the poles [14]. The absence of an acentrosomal component in spindle formation is also supported by the obvious absence of the typical Ran-GTP/TPX2-associated Aurora A pathway, as there appears to be no TPX2 ortholog and the only Aurora kinase, AurK, has more functional similarity to Aurora B [15]. Moreover, the adapter complex for the nucleation of microtubules at kinetochore microtubules, Augmin, is missing in the *Dictyostelium* genome as well.

## 2. The Centrosome and Its Linkage to the Nucleus

**Figure 2:**
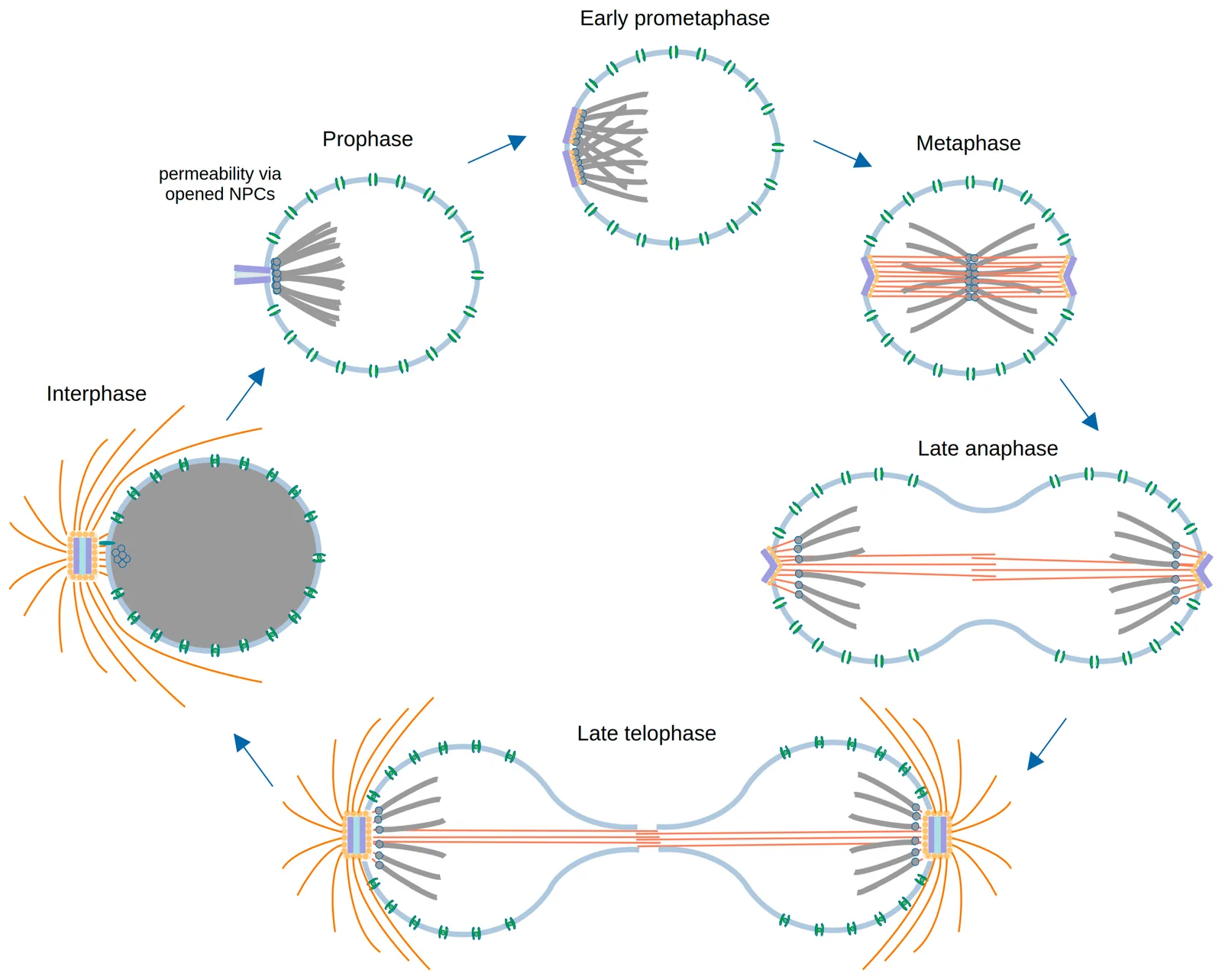
Mitotic events at the nuclear envelope, centrosomes, and chromatin in *Dictyostelium*. The nuclear envelope is shown in light blue, chromatin and centromeres/kinetochores are in gray, microtubules are in orange, and nuclear pore complexes are in green. The centrosome with its core structure with three major layers and the corona with the microtubule nucleating nodules are schematically drawn. Note that nuclear pore complexes partially disassemble during prophase and reassemble in telophase. The figure is in part taken from ref. [10]. See the main text for details.

The *Dictyostelium* centrosome has no centrioles but consists of a cylindrical multilayered structure (known as the core structure) with three main layers surrounded by a so-called corona (Figure 1). The corona is the equivalent of the pericentriolar matrix of animal centrosomes [16]. As such, it contains proteins typically involved in microtubule nucleation and organization, including γ-tubulin complex components, an XMAP215 ortholog (CP224), a TACC protein, EB1, CDK5RAP2, and CP148 (presumed pericentrin ortholog). In addition, CP248/250 is also found there, for which there is no obvious ortholog in animal cells, although there is some functional and immunological evidence that it could be related to mammalian C-Nap1 [16], a protein involved in the linkage of mother and daughter centrioles [17]. Yet, in *Dictyostelium* it could play a role in centromere function since a C-terminal fragment of the protein is localized to the centromere cluster when expressed as a GFP-fusion protein [18]. The layered core structure is also well characterized [16,19], but not particularly relevant in the context of this review. Upon entering mitosis, the corona dissociates, and all microtubules are temporarily lost (Figure 2).

During this process, the core structure enlarges, and a fenestra forms in the nuclear envelope, into which the mitotic centrosome, now consisting only of the core structure, enters. The middle layer of the three main layers of the core structure disappears, and the remaining outer layers, which predominantly consist of Cep192 [19], separate within the persisting nuclear envelope and form the mitotic spindle poles [13,20]. Permeability for spindle assembly factors is achieved by partial disassembly of nuclear pore complexes [12,13]. During this semi-closed mitosis, a central microtubule spindle forms between the diverging poles, and microtubule connections to the kinetochores develop. During the second half of mitosis, the plaque-like spindle poles fold, recreating the multi-layered structure. In late mitosis, the mitotic centrosomes leave the nuclear envelope, and the opening is closed again. The presence of ESCRT proteins at the closure site suggests that the ESCRT complex is involved in this process [13,21]. The connecting structure between the centrosome and the nuclear envelope is reminiscent of the LINC complex, as it contains Sun1 [8,9]. Sun1 is located in both nuclear envelope membranes and is concentrated in the pericentrosomal region. It also co-purifies with isolated centrosomes, suggesting that it interacts directly with one or more centrosome components [9]. On the nuclear side, the centrosome–nucleus connector is also linked to the centromere cluster, in which all six centromeres are densely packed [22] (Figure 1). The *Dictyostelium* lamin NE81 may be involved in this process as well, as it interacts with Sun1 [23].

## 3. Mitotic Microtubule Organization

As mentioned above, due to the absence of the typical players in Ran-dependent acentrosomal spindle organization, such as TPX2, Augmin, and Aurora A [15], it is likely that the mitotic centrosomes serve as the only microtubule organizers in *Dictyostelium*. Yet, mutational analysis of CP148 and CDK5RAP2 revealed interesting differences in mitotic microtubule organization during interphase and mitosis. Both proteins are components of the corona. While CDK5RAP2 (also called Cep161 in *Dictyostelium* [24]) is a true ortholog of the protein of the same name in mammals, CP148 is a likely ortholog of pericentrin/kendrin/Spc110 [16]. Both proteins usually act as binding partners and organizers of γ-tubulin complexes (γTuC). However, when CP148 is depleted via RNAi, cells are unable to organize microtubules during interphase but are perfectly able to organize normal bipolar mitotic spindles, which also explains why the RNAi strain is viable [25]. Thus, CP148 is dispensable for spindle assembly, which is in line with the observation that CP148 dissociates at the onset of centrosome duplication during loss of the corona and remains largely absent from mitotic centrosomes until telophase. In contrast, CDK5RAP2 is absent from centrosomes only very briefly at the time point of corona disintegration but quickly re-associates with the duplicating centrosomes at the transition from prophase to prometaphase, i.e., when the microtubule connections between the spindle poles and kinetochores become established [26]. This scenario largely phenocopies the behavior of γ-tubulin during mitosis [20]. Furthermore, cells with RNAi depletion of CDK5RAP2 not only display disorganized microtubules during interphase (similar to the CP148 RNAi strain) but also show impaired spindle organization [26]. Taken together, this is in agreement with the idea that γTuCs are anchored to the spindle poles via CDK5RAP2.

## 4. Centromeres and Kinetochores

The six chromosomes of haploid *Dictyostelium* amoebae are telocentric. Telomeres and centromeres are therefore immediately adjacent [27]. The centromeric DNA consists mainly of repetitive methylated sequences of the retrotransposon DIRS-1, and centromeric nucleosomes exhibit a high concentration of K3K9me2/3 modifications [22,28,29,30,31]. Centromere- and kinetochore-associated proteins in *Dictyostelium* are listed in Table 1.

The first identified centromere markers were the two isoforms of heterochromatin protein 1 (HP1) (HcpA and HcpB). When they were expressed as GFP-fusion proteins, both co-localized at the centromere cluster, which was also characterized by histone H3 lysine 9 dimethylation [22]. During metaphase, up to six independent fluorescent spots were discernible in conventional fluorescence microscopy, which nicely corresponds with the number of chromosomes.

**Figure 3:**
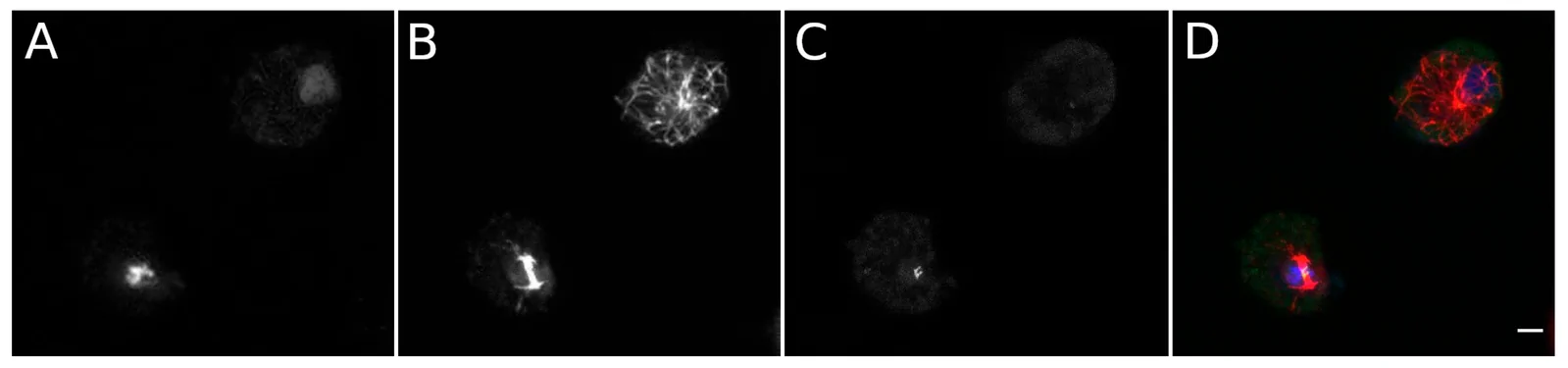
Localization of GFP-Spc24 during interphase (upper cell) and metaphase (lower cell). *Dictyostelium* cells expressing GFP-Spc24 (**C**); green cells in subfigure (**D**) were fixed with glutaraldehyde and stained with anti-α-tubulin antibodies (**B**); red in (**D**). DNA is stained with DAPI (**A**); blue in (**D**). Maximum intensity projections of deconvolved z-stacks are shown. The merged image is shown on the right and the green channel only on the left. Bar = 2 µm. Modified from ref. [39].

As in other organisms, nucleosomes in the centromere region are characterized by the presence of a specific H3 isoform, called CenH3 in *Dictyostelium* [28]. CenH3 is also the only known centromere-specific histone and is thought to have the same function as CENP-A and/or CENP-T (see below). Moreover, Cenp68 has been established as a useful marker for centromeres [18,35]. In addition, only recently, cyclin-dependent kinase 1 (CDK1) and cyclin B were found to be associated with centromeres during G2 and mitosis [15]. During the assembly of kinetochores, the spindle assembly checkpoint component Mad1 can be used as an additional marker [18,40] together with the Ndc80 complex [1]. GFP strains exist for three of the four components of the latter [39], i.e., Nuf2 (microtubule-binding side of the complex) and Spc24/Spc25 (centromere-oriented side of the complex). The staining pattern, as shown in Figure 3, with a strong GFP-staining at kinetochores during mitosis and an absent localization of the fusion proteins during interphase, is also representative for Spc25 and Nuf2.

In addition, INCENP and AurK (the only Aurora kinase in *Dictyostelium*) were identified as markers for the chromosomal passenger protein complex at kinetochores during mitosis [32,38] as well as the polo-like kinase Plk, the centrosomal proteins CP248, EB1, CP224 and TACC [15,18,33,35,37]. The latter three are also known as microtubule-plus-end tracking proteins, which explains their localization at the kinetochores. Plk is likely required for regulation of the Bloom’s syndrome helicase complex to ensure centromere stability during mitosis [41], since major players of this pathway such as Blm and Pich are present in the *Dictyostelium* genome (dictybase.org). Otherwise, the centromere/kinetochore complex, which is well described in animals and yeasts, is still characterized insufficiently in *Dictyostelium*. In addition to the proteins mentioned above, the *Dictyostelium* genome project reveals possible orthologs for CENP-I (Mis6 in yeast; DDB_G0291285) and KNL-1/Spc7/Spc105 (DDB_G0283381). CENP-I is a linker between CENP-A (centromere-specific histone 3) and the KMN network, consisting of KNL-1, the MIS-12 complex (MIND in yeast), and the NDC-80 complex [42]. No ortholog could be identified for any of the subunits of the MIS-12 complex of animals or yeasts (MIND). Yet, we cannot exclude that such orthologs remain unidentified due to too-low sequence similarity. Orthologs of the animal outer centromere proteins CENP-C, CENP-N, and CENP-O/P/Q/U appear to be absent as well. The only centromere-specific H3, CenH3, is significantly elongated at the N-terminus compared with the ubiquitous CENP-A protein [28]. Thus, it is reminiscent of CENP-T. As CENP-A is more ubiquitously present in all eukaryotes and CenH3 is the only centromere-specific histone in *Dictyostelium*, it is possible that it fulfills the role of both CENP-A and CENP-T.

## 5. Mutations Affecting Centromere Clustering

Some of the centromeric proteins mentioned above are also involved in the centrosome–centromere linkage and are good candidates for a role in centromere clustering (Table 2).

Cenp68 has been employed as a useful centromere marker for live cell imaging of centromere behavior in several studies. Cenp68 imaging showed that the centromere cluster disperses during prophase and the centromeres of the six chromatid pairs become individually distinguishable, even though they remain closely together. Individual centromeres are still visible in the daughter nuclei in anaphase, but in telophase they merge again into a cluster that remains associated with the centrosome [35,46]. Mutants with dominant-negative effects on the function of Sun1 (overexpression of GFP-Sun1 or GFP-ΔSun1) not only showed a drastic increase in the distance between the centrosome and the nucleus but also caused the centromere cluster to be positioned arbitrarily at the nuclear envelope while remaining together as a cluster [9]. The same phenotype was observed upon knockout of the unconventional kinesin Kif9 [45]. This membrane-bound kinesin of the outer nuclear membrane (ONM) appears to contribute to positioning the centrosome close to the nucleus through its microtubule depolymerizing function. Furthermore, knockout of Kif9 resulted in a more even distribution of Sun1, which is normally concentrated in the pericentrosomal region [9]. Taken together, these findings point to a possible interaction of Kif9 with Sun1. However, direct evidence for this assumption is still lacking. In contrast to the canonical LINC complex, the centrosome–nucleus connecting structure does not appear to contain a classic KASH domain protein in the ONM, as no gene encoding a KASH domain has yet been identified in the *Dictyostelium* genome. Consequently, no KASH domain is found in Kif9 or in interaptin, which is otherwise similar to the mammalian KASH domain protein nesprin [43]. Although interaptin is located at the nuclear envelope, it does not interact with Sun1 [8] and has no known role in the centrosome–nucleus linkage (Figure 1). Unlike in other organisms studied, Sun1 is not present exclusively in the inner nuclear membrane (INM) but occurs to the same extent in the ONM [9]. However, after it was shown that Sun1 trimers can also form head-to-head hexamers with each other [47], it is also conceivable that Sun1 is the only membrane protein in the connecting structure (review in ref. [10]).

In analogy to animal cells, *Dictyostelium* dynein probably plays a role in establishing the centrosome–nucleus connection, since a hypomorphic mutation of the dynein regulator LIS also led to an increase in the distance between the centrosome and the nucleus [44]. A possible effect on the positioning of the centromere cluster was not investigated in this study. Furthermore, disruption of the centrosome–nucleus linkage was observed upon knockdown of the pericentrin-like centrosome protein CP148 by RNAi [16,25]. Interestingly, in addition to the microtubule organization phenotype described above, this also resulted in the dispersion of the centromere cluster.

Although the role of CP148 in the centrosome–nucleus connection is still unclear, its presence could indicate that Sun1 interacts with proteins of the corona in the ONM. This is also supported by experiments in a strain carrying a mutation in the corona component CP248/CP250, which exhibited an even distribution of Sun1 throughout the nuclear envelope, i.e., the pericentrosomal concentration was abolished, similar to the Kif9 knockout [36].

## 6. Significance of Centromere Clustering

As mentioned above, centromere clustering, including the so-called Rabl configurations, in which telomeres are also closely grouped together, has been observed in numerous organisms. Although an Rabl configuration is not observed in *Dictyostelium*, as telocentric chromosomes are present there [27], the molecular basis and significance may be the same. The situation in *Dictyostelium* is very reminiscent of that in *Schizosaccharomyces pombe*, where the spindle pole body (yeast centrosome) is associated with the cytosolic side of the nucleus, and centromeres are also clustered next to the spindle pole body [48]. Here, the canonical LINC complex connects the spindle pole body across the nuclear envelope to the centromere cluster [49]. It was originally assumed that the formation of the centromere cluster was mediated by the inner nuclear membrane protein Ima1 together with the Ndc80 complex of the outer kinetochore [50]. However, a later study showed that Ima1 does not play a role in the formation of centromere clusters in *S. pombe* [51]. The *Dictyostelium* ortholog of Ima1 (encoded by DDB_G0292450) also localizes to the nuclear envelope as a GFP fusion protein. However, since it is never concentrated at the centromeres (own unpublished observations), the idea that Ima1 could be involved in centromere clustering in *Dictyostelium*, as it was originally proposed in *S. pombe*, was refuted. Now, it is supposed that centromere clustering in *S. pombe* essentially depends on Csi1, whereby Csi1 is associated with the Sun1 ortholog Sad1 and the HeH/LEM domain protein Lem2 at the centromeres [52,53]. On the centromere side, Csi1 is directly or indirectly connected to the outer and inner kinetochore components Nuf2 (Ndc80 complex) and Mis6, respectively [4,52]. Temperature-sensitive mutants of the latter two proteins showed a dissolution of the centromere cluster [54,55]. In *S. pombe*, it was shown that centromere clustering in interphase is independent of microtubules [56]. In *Dictyostelium* as well, there is no evidence for the involvement of microtubules in centromere clustering, as tubulin is not present on the nuclear side of the connecting structure during interphase. Another possible candidate for a role in centromere clustering in *Dictyostelium* could be a HeH/LEM domain protein. The only homolog of this protein family, the INM protein Src1, is concentrated at the spindle poles during telophase, i.e., at the time when the centromere cluster reforms [21,57]. Despite the parallels to fission yeast, there are a number of obvious differences in *Dictyostelium*. For example, no ortholog to Csi1 could be identified in the *Dictyostelium* genome, and the kinetochore proteins Mad1, Nuf2, Spc24, and Spc25 were not found at the centromere cluster in interphase cells [18,39], strongly indicating that, as in animal cells, the kinetochores are assembled only at the G2/M transition. As mentioned in the overview (see Section 1), centromere clustering could highly increase the probability that freshly assembled kinetochores establish correct connections to the duplicating centrosomes/spindle poles in the absence of a Ran-driven acentrosomal component in spindle organization.

## 7. Putative Function of Cenp68

**Figure 4:**
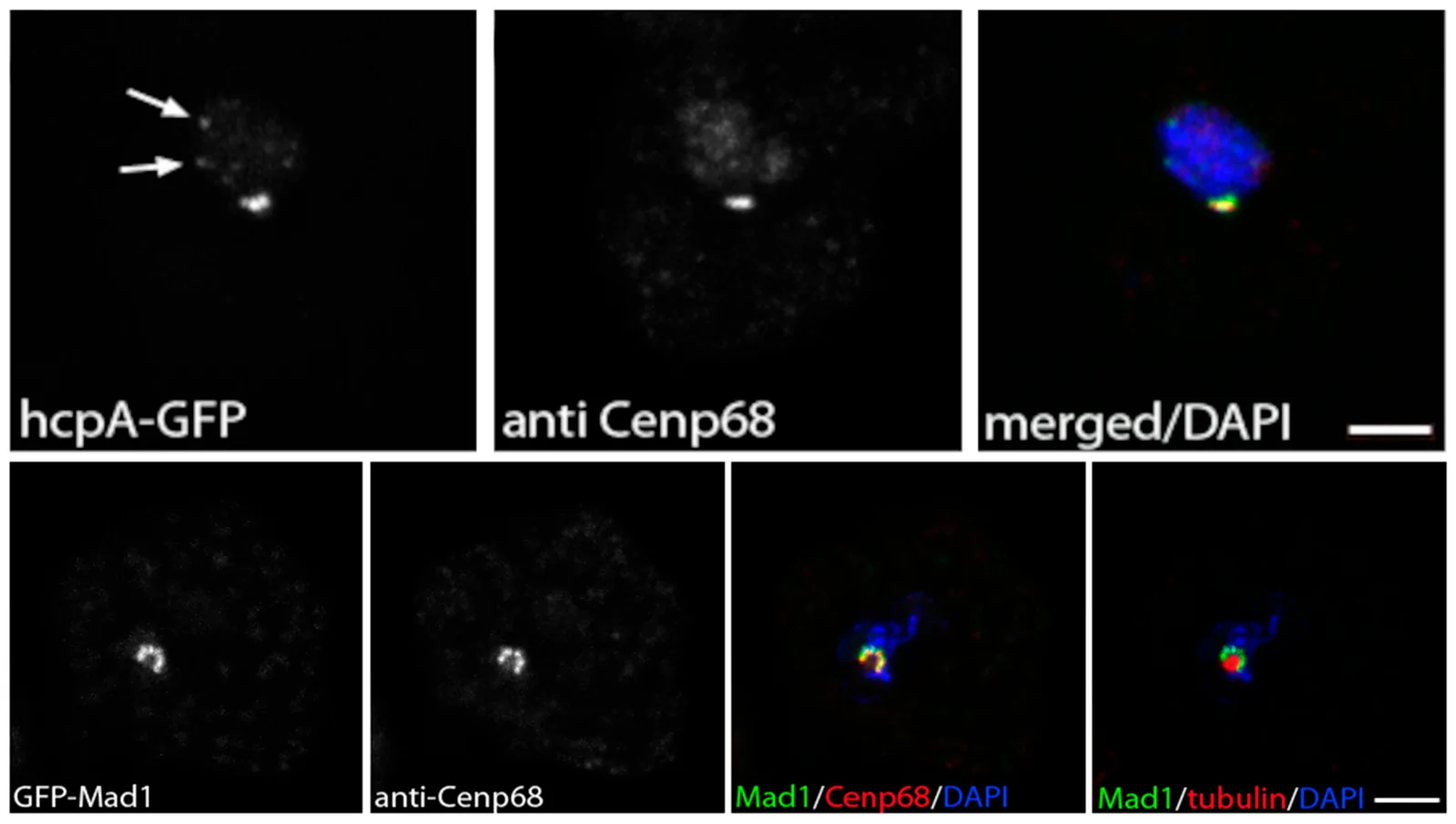
Localizations of endogenous Cenp68, HP1-GFP and GFP-Mad1. *Dictyostelium* cells expressing HP1 (isoform hcpA) GFP (**upper panel**) or GFP-Mad1 (**lower panel**) were fixed with glutaraldehyde and stained with anti-Cenp68 (red) and anti-α-tubulin antibodies (red), respectively, as indicated in the figure. DNA was stained with DAPI (blue). The upper panel shows a typical interphase, and the lower panel a typical prophase. HP1 is also found in lower intensity spots elsewhere in the nucleus (arrows). Widefield microscopy: maximum intensity projections of deconvolved z-stacks are shown. Bars = 2 µm. Figures taken from ref. [40].

Cenp68, which was already mentioned as a useful centromere marker, is still poorly characterized at the functional level. Cenp68 was originally found in a proteome project to identify proteins in purified centrosomes [18]. This was not surprising due to the known close linkage of centrosomes and centromeres during interphase [58]. As already mentioned, overexpression of Cenp68 as a GFP fusion protein showed strong staining of the centromere cluster throughout the cell cycle, which was confirmed for the endogenous protein in stainings with a polyclonal antibody generated against Cenp68 in strains expressing either hcpA-GFP (HP1 isoform A) or GFP-Mad1 [35,40] (Figure 4).

Initially, a weak primary sequence similarity suggested that Cenp68 could be an ortholog of yeast Bbp1p, a permanent resident of the *S. cerevisiae* spindle pole body (SPB). At the SPB, Bbp1p plays an important role in SPB duplication and, as a part of the so-called half-bridge, in tethering the central plaque of the SPB to the nuclear envelope [59]. Yet, a knockout of Cenp68 in *Dictyostelium* was viable and had no striking phenotype and, importantly, it had no effect on tethering the centrosome to the nucleus [40]. Since the cells are haploid, it is also clear that in the AX2 laboratory strain, Cenp68 is not an essential protein. A search for binding partners for GFP-Cenp68 using mass spectrometry yielded Prpf4B (Pre-mRNA Processing Factor 4 homolog B, DDB0216281) as the only possible match, but this protein does not show any concentration at the centromere cluster of *Dictyostelium* when expressed as a GFP fusion protein [40]. However, recently, advanced artificial intelligence-based protein structure predictions (AlphaFold 3) led to a new hypothesis for a function of this protein, as Cenp68 also shows distant sequence similarity to Csm1 of *S. cerevisiae*, a protein related to Spc24/25, and a part of the monopolin complex [60].

**Figure 5:**
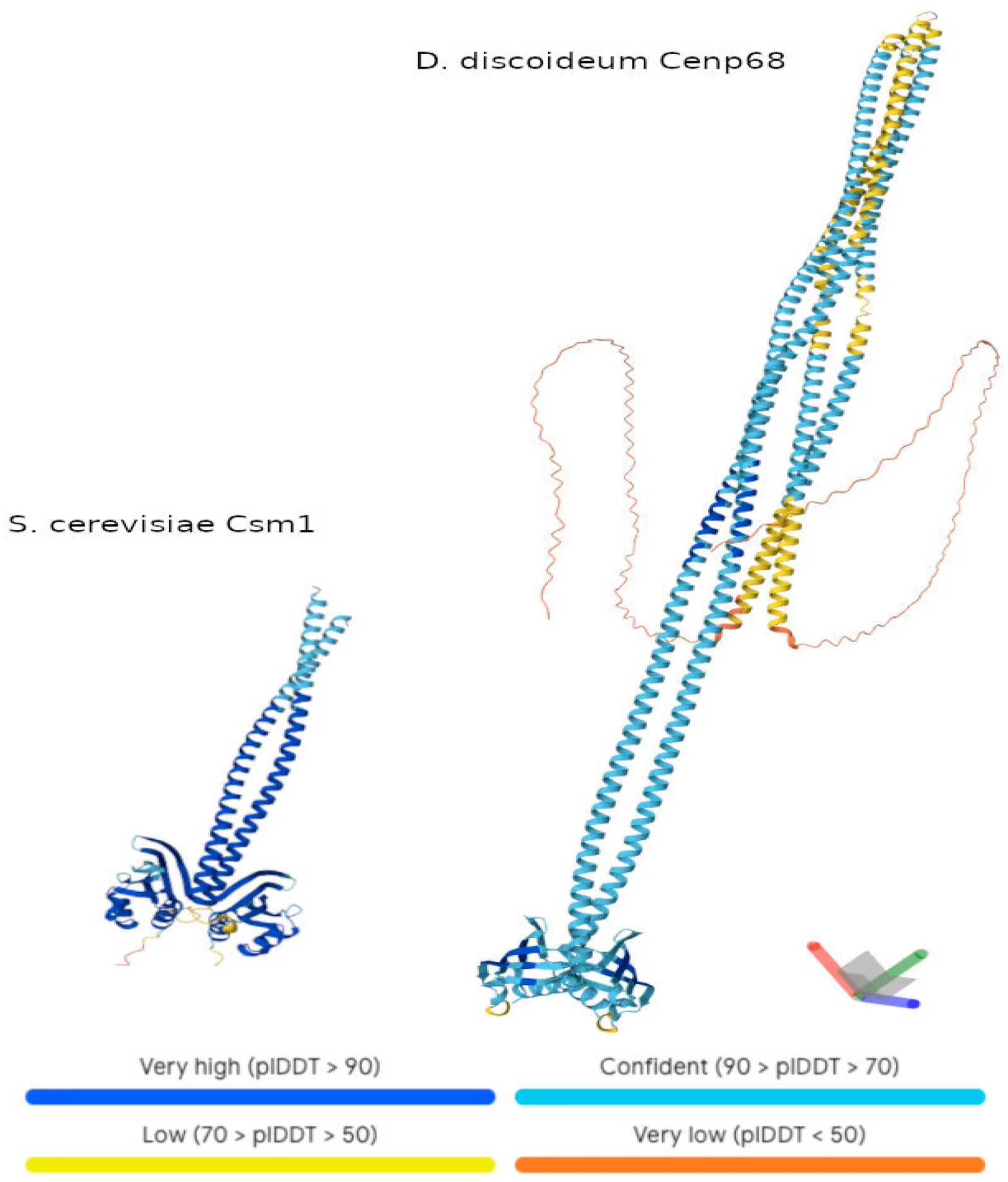
Structural predictions of Csm1 (**left**) and Cenp68 (**right**) dimers. Models were calculated using AlphaFold 3 [61]. The color code for the pIDDT (local per-atom confidence score) is given on the bottom. Predicted template modeling values for Csm1 are pTM = 0,73 and for Cenp68 pTM = 0.63, for the corresponding C-terminal half (pTM = 0.34 for the whole sequence including the N-terminal partially unfolded part).

In budding yeast, the monopolin complex consists of Csm1/Lrs4 (=Pcs1/Mde4 in *S. pombe*), in which two Csm1 dimers join two Lrs4 subunits to form a V-shaped complex with a 4:2 stoichiometry [60]. Interestingly, AlphaFold 3 analysis of Cenp68 shows a high degree of structural similarity to Csm1, especially in the C-terminal half of the *Dictyostelium* protein containing the globular domains. This supports the idea that Cenp68 and Csm1 may indeed be orthologs (Figure 5). In both cases, AlphaFold predicts a dimer that is held together by a long α-helical coiled coil domain that ends with a C-terminal globular domain. The latter appears very similar between both species, with three β-sheets and three short α-helices. The main difference is that the N-terminal α-helical coiled coil domain is much longer in the *Dictyostelium* protein (575 vs. 190 amino acids in Csm1). We could not identify a putative ortholog of Lrs4/Mde4 in *Dictyostelium*; however, its function may be compensated by the extended α-helical N-terminal domain of Cenp68. In *S. pombe*, monopolin is responsible for the mono-orientation of centromeres/kinetochores during meiosis. In principle, *Dictyostelium* amoebae are capable of sexual reproduction. However, since the laboratory strain *Dictyostelium* AX2 does not form macrocysts, it does not have a sexual cycle and therefore does not undergo meiosis. This could explain why the Cenp68 knockout in AX2 did not exhibit an obvious mutant phenotype. However, this raises the question of why Cenp68 is expressed in haploid vegetative cells at all. One appealing idea is that of a novel mechanism in which Cenp68 mediates monopolar orientation of mitotic centromeres. This would make perfect sense since centrosome splitting into two entities, assembly of kinetochores, and appearance of strong tubulin staining at the centrosome/centromere cluster all take place at the same time. Mono-orientation of centromeres/kinetochores could facilitate the establishment of correct microtubule connections between kinetochores and duplicating centrosomes and thus ultimately accelerate spindle formation. Since the duration of mitosis is relatively short, lasting about ten minutes compared with the complete cell cycle of eight to nine hours, a prolongation of prophase and prometaphase by even a factor of two in the Cenp68 knockout strain may have gone unnoticed, as only overall growth rates were analyzed.

## 8. Hypothesis

**Figure 6:**
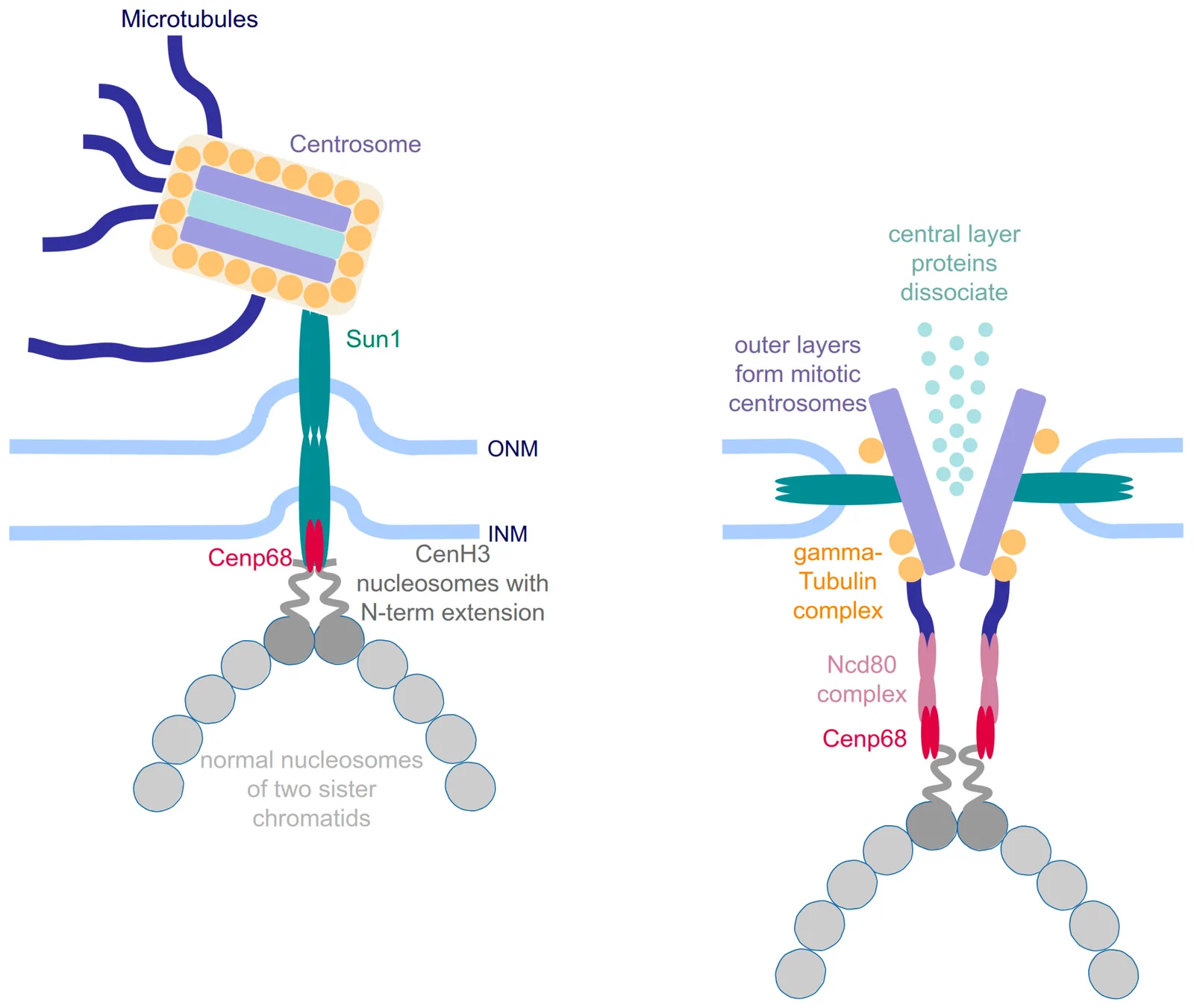
Proposed centrosome–centromere organization during interphase (**left**) and prophase (**right**). During interphase, Sun1 may also interact directly with centromeric chromatin via binding to histones. The so far uncharacterized orthologs of Spc7 and CENP-I, which may associate with the kinetochore complex, are omitted here.

Taken together, our hypothesis of centrosome–centromere organization during interphase and mitosis is as follows (Figure 6). During interphase, Sun1, which binds to the centrosome on the cytosolic side and the centromere cluster on the nuclear side, is present in both nuclear membranes and links both complexes via self-interaction. Sun1 binding to the centromeric chromatin may occur via interactions with histones as in *S. pombe* [62].

As the centromere cluster (labeled by the HP1 isoform hcpB) remained linked to the centrosome in Cenp68 knockout cells [40], Cenp68 itself is not required for the linkage. But, according to our hypothesis, it enables mono-orientation of all centromeres toward the linkage site. During prophase, upon centrosome duplication, the presumed linkage between Sun1 in the INM and Cenp68 is replaced by a linkage between Cenp68 and the Ndc80 complex that may also include the so far uncharacterized orthologs of Spc7 and CENP-I.

Future research will aim at answering the intriguing question of how this transition occurs and is regulated. Further investigation of the exact composition of the centromere cluster and its relationship to the centrosome will provide a better understanding of the evolution of spindle formation strategies in eukaryotic mitosis and may shed light on the extent to which similar strategies have evolved independently.

## Figures and Tables

**Table 1:** Centromere and kinetochore-associated proteins in *Dictyostelium*.

Refs.	Kinetochore (Mitosis)	Centromere (Interphase)	Protein	Gene
[15,32]	+	−	Aurora, AurK, AurB	*aurK*
[33,34]	+	−	CP224, DdCP224, XMAP215-family	*cepJ*
[35]	+	−	TACC	*tacc*
[18,36]	+ *	+ *	CP248, CP250	*cepK*
[15]	+	+	CDK1	*cdk1*
[15]	+	+	Cyclin B	*cycB*
dictybase.org	ND	ND	Mad2	DDB_G0282747
dictybase.org	ND	ND	Knl1, Spc7, Spc105	DDB_G0283381
dictybase.org	ND	ND	Cenp-I, Mis6	DDB_G0291285
[18,35]	+	+	Cenp68	DDB_G0293620
[37]	+	−	EB1	*eb1*
[22]	+	+	HcpA (HP1 isoform A)	*hcpA*
[22]	+	+	HcpB (HP1 isoform B)	*hcpB*
[28]	+	+	DdCenH3, CenH3	*h3v1*
[38]	+	ND	INCENP	*icpA*
[18]	(+)	+	Mad1	*mad1*
[39]	+	−	Ndc80	*ndc80*
[39]	+	−	Nuf2	*nuf2*
[15]	+	−	Plk	*plk*
[39]	+	−	Spc24	*spc24*
[39]	+	−	Spc25	*spc25*

* Detected only for the C-terminal half of the protein. Other kinetochore-associated proteins involved in spindle checkpoint regulation, chromosome segregation, mitotic exit, and centromere stability during mitosis are not within the subject of this review and are therefore not listed in the table. These include Mps1, Rod, CENP-E (kinesin KIF10), Bub1, Bub2, and Bub3, Pich and Blm, for which orthologs are annotated at dictybase.org. ND = not determined.

**Table 2:** Proteins associated with the centrosome–centromere linkage.

Refs.	Nuclear Side	Cytoplasmic Side	Protein	Gene
[8,9]	+	+	Sun1	*sun1*
[43]	−	+	Interaptin	*apbD*
[25]	−	+	CP148	*cepG*
[44]	−	+	Lis1	*lis1*
[45]	−	+	Kif9	*kif9*
[11]	+	−	Lamin, NE81	*lmnB*
[36]	+	+	CP248, CP250	*cepK*

## Data Availability

Genomic data on the genes are available at http://dictybase.org (accessed on 9 August 2026). No new data were created or analyzed in this study. Data sharing is not applicable to this article.

## Abbreviations

The following abbreviations are used in this manuscript:

INM	Inner nuclear membrane
ONM	Outer nuclear membrane
ESCRT	Endosomal sorting complex required for transport
LINC	Linker of the nucleus and the cytoskeleton
